# Identification of the Moving Junction Complex of *Toxoplasma gondii*: A Collaboration between Distinct Secretory Organelles

**DOI:** 10.1371/journal.ppat.0010017

**Published:** 2005-10-21

**Authors:** David L Alexander, Jeffrey Mital, Gary E Ward, Peter Bradley, John C Boothroyd

**Affiliations:** 1 Department of Microbiology and Immunology, Stanford University, Stanford, California, United States of America; 2 Department of Microbiology and Molecular Genetics, University of Vermont, Burlington, Vermont, United States of America; 3 Department of Microbiology and Immunology, University of California, Los Angeles, Los Angeles, California, United States of America; Northwestern University Medical School, United States of America

## Abstract

Apicomplexan parasites, including *Toxoplasma gondii* and *Plasmodium sp*., are obligate intracellular protozoa. They enter into a host cell by attaching to and then creating an invagination in the host cell plasma membrane. Contact between parasite and host plasma membranes occurs in the form of a ring-shaped moving junction that begins at the anterior end of the parasite and then migrates posteriorly. The resulting invagination of host plasma membrane creates a parasitophorous vacuole that completely envelops the now intracellular parasite. At the start of this process, apical membrane antigen 1 (AMA1) is released onto the parasite surface from specialized secretory organelles called micronemes. The *T. gondii* version of this protein, TgAMA1, has been shown to be essential for invasion but its exact role has not previously been determined. We identify here a trio of proteins that associate with TgAMA1, at least one of which associates with TgAMA1 at the moving junction. Surprisingly, these new proteins derive not from micronemes, but from the anterior secretory organelles known as rhoptries and specifically, for at least two, from the neck portion of these club-shaped structures. Homologues for these AMA1-associated proteins are found throughout the Apicomplexa strongly suggesting that this moving junction apparatus is a conserved feature of this important class of parasites. Differences between the contributing proteins in different species may, in part, be the result of selective pressure from the different niches occupied by these parasites.

## Synopsis

Among the world's most important pathogens are a group known as the Apicomplexa. These are single-celled, eukaryotic parasites that cause a range of diseases including malaria and some AIDS opportunistic infections, such as toxoplasmosis and cryptosporidiosis. The group shares several properties: first, they are all intracellular parasites that require a host cell in which to grow; second, they all have an extraordinary collection of structures at their front end, the eponymous apical complex; and third, during invasion, each forms an intimate association with the host cell surface. This ring of contact, which migrates down the parasite as invasion proceeds, is termed the moving junction (MJ). Until now, the composition of the MJ has been a complete mystery. Here, the authors identify four proteins that apparently make up the MJ in *Toxoplasma gondii* and show that the structure is apparently conserved throughout the Apicomplexa, including in the malaria parasites. Surprisingly, forming the MJ appears to be a collaboration between two, completely different secretion organelles within the apical complex. Detailed study of the MJ complex will shed light on what adaptations each parasite has evolved for the hosts and the cell type they infect. It may also represent an important target for prevention and treatment.

## Introduction

*Toxoplasma gondii* is an obligate intracellular parasite that belongs to the phylum Apicomplexa. All members of this phylum exhibit a similar process for invading into host cells [1]. The process begins with gliding motility that utilizes a reversible attachment to migrate over the surface of the host cell, perhaps to find a susceptible site for entry. The parasites then establish an intimate association, involving reorientation to put the apical secretory structures in contact with the host membrane [2]. This reorientation is coincident with a transient spike in host plasma membrane conductance, consistent with a break in the bilayer [3]. Next, the specialized secretory organelles in the parasite's apex, called micronemes and rhoptries, secrete their contents [1,4]. A moving junction (MJ), where the host and parasite plasma membranes are in intimate contact, is then formed.

The MJ is associated with a visible constriction around the invading parasite that migrates from anterior to posterior end during invasion. This results in the creation of a parasitophorous vacuolar membrane (PVM) derived from invagination of the host plasma membrane [3–6]. The entire process is highly orchestrated and driven by the parasite's actin and myosin machinery [7–11].

Among other functions, the MJ apparently serves to exclude most host membrane proteins from entering the PVM [4]. Transmission electron micrographs of invading *Plasmodium* merozoites and *Toxoplasma* tachyzoites have shown the MJ to be associated with electron-dense structures reminiscent of tight junctions, but the composition of these is not known [5,6]. Numerous proteins involved in invasion have been identified, but only one of these, MCP-1 of *P. falciparum*, has been shown to be associated with the MJ [12,13]. This protein has no signal peptide or transmembrane domain and is presumed to interact with the MJ complex from the inside of the parasite; hence the key surface molecules that form this complex have remained a mystery.

Apical Membrane Antigen 1 (AMA1) is among the proteins directly implicated in host cell invasion by several members of the phylum, including *Toxoplasma*, *Plasmodium,* and *Babesia* [14–16]. AMA1 is unique to the Apicomplexa and has been localized to the micronemes of developing intracellular parasites and to the apical surface of extracellular parasites just prior to invasion [17–19]. During invasion, AMA1 migrates across the tachyzoite surface from where it is proteolytically shed in a soluble form [18–21]. These observations have led to the hypothesis that AMA1 is one of the parasite adhesins that transiently engage host receptors during the invasion process [22]. This model is supported by the observation that anti-AMA1 antibodies block invasion at a stage following the initial attachment to the host plasma membrane in *Toxoplasma* [18], *Plasmodium* [2] and *Babesia* [16]. As predicted from these data, TgAMA1 is an essential gene, and conditional knockout parasites are significantly impaired in their ability to invade [23].

TgAMA1 and its homologues from other Apicomplexa have the structural characteristics of type-I transmembrane proteins. The ectoplasmic region contains 16 invariant cysteine residues that are present in all AMA1 sequences, indicating a conserved overall topology among the homologues [24,25]. AMA1 from *P. vivax* has been crystallized, and analysis of the structure suggests a receptor-binding role in invasion, requiring inter-domain interaction [26].

To better understand the precise role of TgAMA1, we sought to identify the parasite proteins with which it associates. We describe here three such proteins, at least two of which, RON2 and RON4, surprisingly, derive from the rhoptry necks, not the micronemes, from which AMA1 is released. At least one, RON4, is secreted upon attachment to host cells, where it then precisely and predominantly localizes to the MJ as it migrates down the length of the invading parasite. In wild-type parasites, only a small amount of TgAMA1 is associated with RON4 at the MJ, but in engineered parasites with reduced amounts of TgAMA1 this proportion rises and a clear concentration of TgAMA1 is evident at the MJ. This crucial complex appears to be conserved across the Apicomplexa.

## Results

### Identification of AMA1-Associating Proteins

To determine the proteins associating with TgAMA1, immunoprecipitation from detergent extracts of extracellular *Toxoplasma* tachyzoites was performed using an independent monoclonal antibody (mAb) recognizing one of two distinct TgAMA1 epitopes. The mAb B3.90 [19] detects an epitope in the ectodomain whereas mAb CL22 [18] was raised to a dodecapeptide at the C-terminus of TgAMA1. Each of these antibodies immunoprecipitated TgAMA1 (~70kDa) and near identical profiles of TgAMA1-associating proteins (AAPs; Figure 1A). These AAPs, with apparent sizes of ~145, 130, 110, and 45 kDa, were stably associated with TgAMA1 at high salt concentrations (500 mM NaCl), suggesting a highly specific interaction (unpublished data). The specificity of the AAP association was further demonstrated by immunoblotting the material that co-precipitated with anti-TgAMA1 and probing for the highly abundant MIC2, GRA7, ROP1, or ROP2/3/4 proteins. None of these proteins were detected (unpublished data).

**Figure 1:**
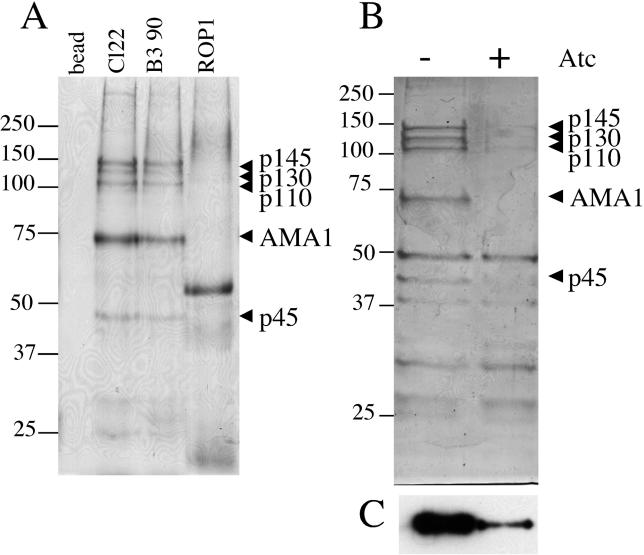
Identification of TgAMA1-Associating Proteins (A) Monoclonal antibodies CL22 and B3.90 were used to immunoprecipitate TgAMA1 and associating proteins from RIPA lysates of wild-type tachyzoites. The resulting material was resolved by SDS-PAGE followed by Coomassie staining to detect the proteins. Controls were immunoprecipitation with beads alone or an irrelevant mAb specific for the rhoptry bulb protein, ROP1. Each lane represents the product of 10^7^ parasites. The polypeptides specifically associating with TgAMA1 are denoted on the right by their estimated molecular masses in kDa: p145, p130, p110, and p45. Molecular mass markers (in kDa) are indicated on the left of each panel. (B) mAb CL22 was used to immunoprecipitate TgAMA1 and associating proteins from *ΔAMA/AMA1-myc* parasites without (−) or with (+)Atc. (C) Immunoblot of the lanes shown in Figure 1B probed with antibody specific for TgAMA1.

Further evidence for the specificity of the AAPs for TgAMA1 was obtained using a *Toxoplasma* line in which the endogenous *TgAMA1* locus has been disrupted and several tetracycline-repressible myc-tagged copies have been introduced (*ΔAMA1/AMA1-myc*; [23]). In the absence of added drug, this parasite line expresses ~10% of the constitutive TgAMA1 levels, which is a sufficient level for essentially wild-type levels of invasion and growth. Tetracycline treatment of these parasites reduces TgAMA1 levels to ~0.25% of wild-type amounts. Although such parasites have no apparent defect in motility or initial attachment to the host cell, they are almost completely blocked in their ability to invade [23]. A specific role for TgAMA1 in invasion is further indicated by the observation that if tetracycline is added to cultures in which the parasites have already entered a host cell, there is no effect on further growth within that cell or on egress, but subsequent invasion is blocked. Immunoprecipitation of the AAPs from the *ΔAMA1/AMA1-myc* line grown in the absence of tetracycline resulted in recovery of the same profile of AAP bands (Figure 1B). As expected from its lower level of expression, markedly less TgAMA1 was recovered on a per-parasite basis, relative to wild-type parasites. For the four AAPs, however, nearly identical amounts were obtained (Figure 1 and unpublished data). As a result, the stoichiometry appeared to go from an excess of TgAMA1 over the AAPs in wild-type parasites to something closer to equal levels in the engineered parasites. This point is discussed further below.

Under tetracycline-repressed conditions, immunoprecipitation with anti-TgAMA1 antibodies yielded much reduced TgAMA1 and the four AAPs (Figure 1B), although all were still detectable by Coomassie staining (Figure 1B) and/or immunoblot (Figure 1C). A variable number of bands below ~50 kDa were also observed in these gels, but apart from AAP45, they were not reproducibly detected in wild-type parasites (Figure 1A) and their abundance was not consistently reduced in the TgAMA1 knock-down conditions [Figure 1B, +Atc (anhydrotetracycline)]. Thus, they were not further pursued.

### Mass Spectrometry-Based Proteomic Analysis of TgAMA1 and the AAPs

To determine the identity of the four AAPs, gel slices from the immunoprecipitation experiments were excised, digested with trypsin, and the eluted peptides were subjected to liquid chromatography-electrospray ionization-ion trap mass spectrometry (LC-MS/MS) analysis (Table 1). This analysis also confirmed that the ~70 kDa band was TgAMA1. Included among the identified peptides for this protein was one with an N-terminal sequence not derived from tryptic cleavage (TgAMA1 amino acids 40–54, Table 1). This likely derives from the pro-peptide cleavage of TgAMA1 described previously [18] and indicates cleavage of 17 amino acids to the C-terminal side of the predicted site for removal of the signal peptide. This N-terminal sequence for mature TgAMA1 was confirmed by Edman sequencing of the same immunoprecipitated TgAMA1 (unpublished data).

**Table 1:**
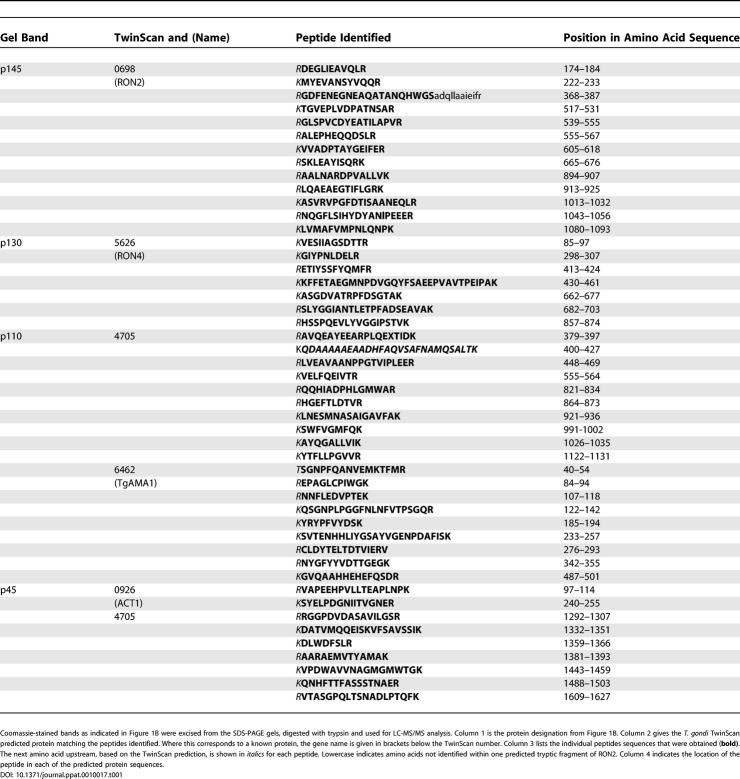
Identification of TgAMA1-Associating Proteins by LC-MS/MS

Mass spectrometric analysis of the three largest AAPs revealed multiple peptides corresponding to *Toxoplasma* TwinScan predicted sequences Ts0698, Ts5626, and Ts4705 for AAP145, AAP130, and AAP110, respectively. Whether analyzed as a total gel slice representing the sizes from ~100 kDa to ~150 kDa, or as individually excised bands, these three predicted proteins and TgAMA1 were the only proteins for which multiple peptides were identified that met the confidence criteria described in the Materials and Methods (Table 1).

Ts0698 corresponds to a protein of ~157 kDa, with a predicted signal sequence and three transmembrane domains. Proteomic analysis of the *Toxoplasma* rhoptries also recently identified this protein [27], and using antibodies to recombinant protein, it was shown to derive from the constricted, anterior portion of the rhoptries termed *rhoptry necks*. Ts0698 was one of four such neck proteins identified and was named RON2. The complete open reading frame was determined by cDNA sequencing; this confirmed the TwinScan prediction of a protein of ~157 kDa. Allowing for removal of a signal peptide, the predicted size (~153 kDa) is slightly larger than that predicted from the mobility on SDS-PAGE (~145 kDa). This leaves open the possibility that it may be processed by proteolytic cleavage to its mature size, a common property of many rhoptry proteins [28] although the discrepancy could also be a result of anomalous migration. There were no apparent functional motifs beyond the signal peptide and three putative hydrophobic transmembrane domains. RON2 is paralogous to two other *Toxoplasma*-predicted proteins, Ts3110 and Ts0430, but none of the peptides detected here exactly match these other two predicted proteins, and so the identity of p145 could be unambiguously assigned to RON2.

Ts5626 corresponds to a protein that TwinScan predicts should be ~78 kDa with a signal peptide and no predicted transmembrane domains. Proteomic analysis of the *Toxoplasma* rhoptries also identified this protein and, using antibodies to recombinant protein showed it to also be a rhoptry neck protein [27]. It was, therefore, dubbed RON4. The complete open reading frame was determined by cDNA sequencing. This indicated that the Ts5626 gene, in fact, corresponds to a predicted protein considerably larger (~107 kDa) than the TwinScan algorithm predicted (the TwinScan prediction miscalled some of the exon/intron boundaries, which is not uncommon). Allowing for removal of a signal peptide, the predicted size (~105 kDa) is still much smaller than that suggested by its mobility on SDS-PAGE (~130 kDa). The retarded migration in SDS-PAGE analysis may be explained by the 44 amino acid repeat sequence and the charges within this repeat [27]. There is a paralogous protein identified in the *T. gondii* database, Ts2928, but none of the peptides detected here exactly match that protein and so the identification of AAP130 as RON4 was unambiguous. No functional motifs, other than a signal peptide, were observed.

Ts4705 predicts a protein of ~179 kDa. This protein was also identified in the rhoptry proteome analysis although neither its size nor its localization were definitively determined [27]. It therefore received no gene name and its true origin remains to be confirmed although all but one of 15 novel proteins that were definitively characterized in the Bradley et al. study proved to be from the rhoptry bulb or neck. It is therefore highly likely that Ts4705 corresponds to a rhoptry protein and given its association with RON2 and RON4 it most likely derives from the rhoptry neck. The discrepancy between the apparent (~110 kDa) and predicted (~179 kDa) sizes for this protein could indicate post-translational processing, as mentioned above for RON2. The identification of Ts4705 peptides in AAP45 is consistent with this possibility (see below) but some of the discrepancy could also be due to abnormal mobility or errors in the TwinScan prediction.

In the excised gel band corresponding to AAP45, two significant peptide hits were identified corresponding to *Toxoplasma* actin (TgACT1), as well as seven peptides from the predicted C-terminus of Ts4705. Immunoblots with antibodies specific to TgACT1 confirmed that actin was present in the TgAMA1-immmunoselected material (unpublished data). Importantly, however and unlike the Coomassie staining of AAP45, there was no difference in the anti-actin signal using TgAMA1-immunoprecipitated material from untreated or tetracycline-treated *ΔAMA1/AMA1-myc* parasites and comparable signals were also seen in preparations obtained by immunoprecipitation using antibodies to an irrelevant control protein, ROP1 (unpublished data). These results strongly argue against a specific association of TgACT1 with TgAMA1. Consistent with this, TgACT1 has been proposed to interact with aldolase [29] and Toxofilin [30] yet no peptides corresponding to either of these two proteins were detected by MS, and neither were detected on immunoblots of material immunoprecipitated with anti-TgAMA1 (unpublished data).

The Ts4705 peptides identified in p45 were all derived from the C-terminal region, with no overlap to peptides identified from p110 (Table 1). This is consistent with a proteolytic processing of the Ts4705 predicted polypeptide into p110 and p45 both of which are associated, directly or indirectly, with TgAMA1.

### Functional Analysis of TgAMA1 and RON Protein Interactions

The finding that the micronemal protein TgAMA1 associates with at least two rhoptry neck proteins was unexpected and begged the question of whether these proteins associate with TgAMA1 in intact parasites or only when given the artificial opportunity of parasite lysis. To determine whether there is an in vivo interaction between the RONs and TgAMA1, therefore, we used chemical cross-linking of live, cultured parasites followed by immunoprecipitation from parasite extracts denatured so as to disrupt most non-covalent interactions (boiled in the presence of detergent). The cross-linker used was the membrane-impermeable, thiol-cleavable 3,3′-Dithiobis (sulfosuccinimidylpropionate) (DTSSP) and the parasites were from a culture in which invasion was synchronized with a high potassium block [31] that was released simultaneously with the addition of the cross-linker. Under these conditions, any proteins that are exposed to the medium and are within twelve angstroms of each other should be cross-linked, including those that are put out onto the surface during invasion.

Following two washes and quenching of any remaining cross-linker, the fibroblast monolayer and attached parasites were lysed and boiled in 1% SDS and then equilibrated into RIPA buffer. Immunoprecipitation with anti-TgAMA1 was performed, as before, followed by DTT reduction and analysis by SDS-PAGE. Immunoblotting revealed RON4 and RON2 specifically associating with significant amounts of TgAMA1 but only if cross-linked before the precipitation (Figure 2A). The specificity of the cross-linking was demonstrated by showing that the control rhoptry protein ROP1 was not co-immunoprecipitated by any of the heterologous antisera and neither did anti-ROP1 antibodies co-precipitate detectable amounts of any of the other three proteins (Figure 2B). Reciprocal experiments using immunoprecipitation with anti-RON4 yielded TgAMA1 (Figure 2C). Some TgAMA1 is detected in the absence of DTSSP, but cross-linking significantly increased the amount of TgAMA1 co-precipitating with RON4. Interestingly, RON2 was pulled down with anti-RON4 regardless of the presence of cross-linker suggesting a covalent or other strong association between these two proteins (Figure 2C). No co-precipitation of TgAMA1 and RON2, or RON4 was seen using heat-denatured lysates from extracellular parasites, with or without prior cross-linking (not shown). These results indicate that an association between TgAMA1 and RONs occurs during the process of invasion and is surface-exposed. That is, the association is not simply an artifact of parasite lysis although this may increase the amount of association that is seen ( Figure 1).

**Figure 2:**
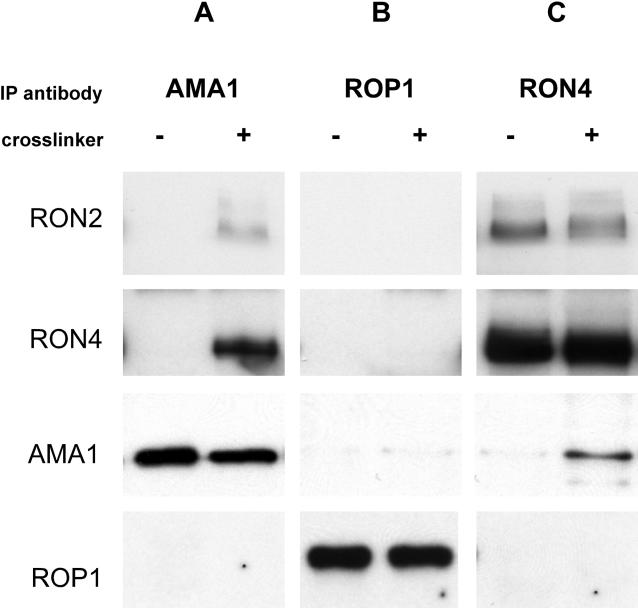
Immunoprecipitation from Lysates of Parasites Subjected to Chemical Cross-Linking in Live Cultures Tachyzoites were allowed to synchronously invade fibroblast monolayers in the absence (−) or presence (+) of the homobifunctional cross-linking agent DTSSP. Invasion and cross-linking were allowed to proceed for 15 min, then the reaction was quenched and invasion stopped. Harvested parasites were extracted in 1% SDS and heat denatured, and then processed for immunoprecipitation with antibodies to TgAMA1 (A), ROP1 (B), or RON4 (C). These immunoprecipitates were separated by reducing SDS-PAGE and analyzed by immunoblotting to determine the presence or absence of RON2, RON4, TgAMA1, or ROP1 in the co-precipitating material.

To explore the association further, tachyzoites that were intracellular and ones that were entering and exiting fibroblast monolayers were analyzed by indirect immunofluorescence (IIF) to identify where in the cell TgAMA1, RON4 and RON2 might be associating. Unfortunately, the antisera available for RON2 gave an IIF signal only using methanol-fixation, and not the formaldehyde-fixation conditions needed to preserve good morphology for IIF. While this did allow identification of RON2 as a rhoptry neck protein [27], no signal was observed on invading parasites indicating that either this protein is released into the medium during invasion or, more likely, the structures it is within are not stabilized with this fixative (unpublished data).

Antibodies to RON4, however, worked well using formaldehyde-fixation and so co-localization with TgAMA1 could be assessed. Using intracellular parasites, and as predicted from the literature, anti-RON4 and anti-TgAMA1 antibodies gave the closely apposed but clearly distinct IIF patterns typical of rhoptry necks and micronemes, respectively (Figure 3). RON4 was also detected in the parasitophorous vacuole (Figure 3, arrows) as previously reported [27]. No TgAMA1 is seen in the vacuolar space. These results confirmed that TgAMA1 and the RON2/4 proteins are initially in distinct compartments within the parasite.

**Figure 3:**
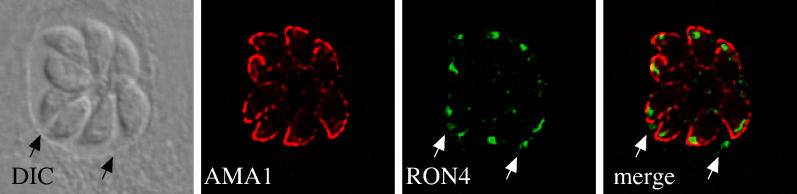
Localization of RON4 and TgAMA1 in Intracellular Tachyzoites DIC and deconvolution processing of IIF were used to image a PV containing eight intracellular parasites that had been fixed with formaldehyde and permeabilized with triton X-100. TgAMA1 was localized with mAb CL22 and Texas-red-conjugated goat-anti-mouse antiserum. RON4 was localized via rabbit antiserum to recombinant RON4 followed by goat-anti-rabbit antiserum conjugated with fluorescein isothyocyante (FITC) (green). The images shown represent an extended focus projection through five 0.1-μm sections after iterative deconvolution. RON4 staining within the vacuole in the regions between the parasites is indicated with an arrow.

### Analysis of AAP Localization in Invading Parasites

The above results predicted that the association of TgAMA1 and RON proteins happens only upon initiation of invasion when the micronemes and rhoptries discharge. To examine this, we used IIF and the invasion-synchronization method described above. At the very start of invasion, antibodies to RON4 can be seen staining the apical tip of parasites that are in intimate contact with the host cell and a very small, circumferential ring (Figure 4A–4D). RON4 is not detected on the surface of unattached parasites (unpublished data). Antibodies to surface antigen 1, SAG1, show bright staining under non-permeabilized conditions and this can be used to identify the portion of an invading parasite outside of the host cell up to the point of contact with the host plasma membrane [32]. Subsequent permeabilization and staining for RON4 shows this protein localizes precisely with the limit of SAG1 staining (Figure 4A–4D). As the parasite enters the host cell, this ring of RON4 staining migrates down the length of the parasite, coincident with the constriction that demarcates the MJ (arrows) between host and parasite (Figure 4E–4H). Fully invaded parasites show a focal staining at the posterior end of the parasite where the PVM appears to contact the host plasma membrane (Figure 4I–4L). There is also some apical staining within the parasite, the intensity of which varies depending on the degree of saponin permeabilization (Figure 4I–4L and unpublished data). The RON4 staining of the MJ, including the final posterior focus is fully apparent in the absence of detergent permeabilization (Figure 4M–4T) consistent with the observation that RON4 is outside the portion of the MJ that keeps antibodies out of the nascent parasitophorous vacuole (PV). The focal surface staining at the site of invasion is only observed within about six min after release from the high potassium block to initiate invasion; after that, and likely when the PV has dissociated from the host plasma membrane [3], this posterior focal staining is no longer detected (unpublished data).

**Figure 4:**
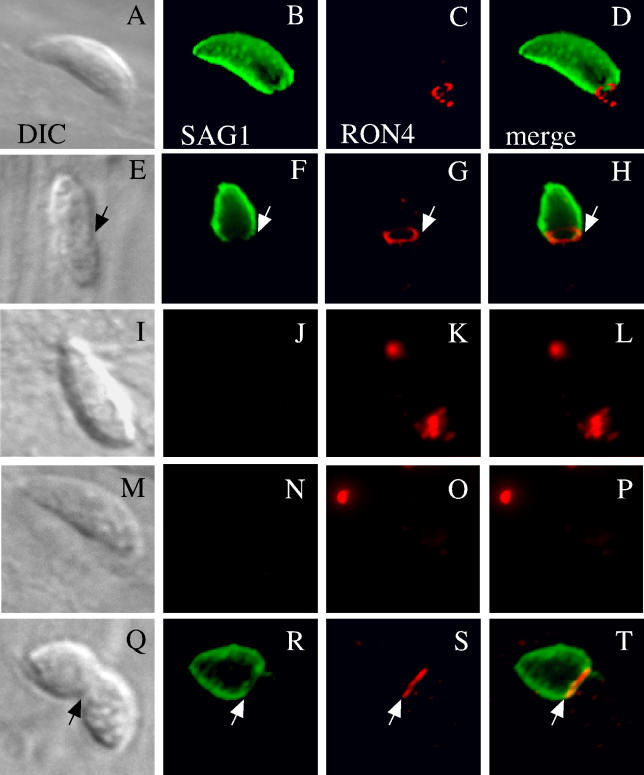
Localization of RON4 During Invasion Tachyzoites were allowed to invade following a potassium shift as described in the text. DIC (A, E, I, M, and Q) and deconvolution IIF were then used to image formaldehyde-fixed parasites as described in Figure 3. The images presented are extended focus projections through ten 0.2-μm sections. Prior to detergent permeabilization, SAG1 was detected with the mAb DG52 followed by goat anti-mouse antiserum conjugated to FITC (green) (B, F, J, N, and R). RON4 detection was with rabbit antisera raised to recombinant RON4 and Texas red-conjugated goat-anti-rabbit antibody; this was done after permeabilization by the addition of saponin (C, G, and K) or without such permeabilization (O, S). The merged images are (D, H, L, P, and T). (A–D) represent a parasite that has just begun invasion as indicated by SAG1 staining over about 80% of the parasite. (E–H) show a parasite about halfway in. (I–L) show a parasite that appears to be fully inside (no SAG1 staining) showing small posterior cap of RON4 and apical staining consistent with rhoptry necks. (M–T) show parasites stained without prior permeabilization. (M–P) also show a parasite fully in, whereas (Q–T) show a parasite at about halfway in. The MJ is indicated by an arrow where it is clearly apparent in the DIC image.

The surface accessibility of RON4 to antibodies suggested that anti-RON4 antiserum might be also inhibitory for invasion, similar to the findings for some antibodies against TgAMA1 and PfAMA1 [2,18,33]. This was tested by pre-incubation of parasites with anti-RON4 antisera and/or inclusion of the antisera upon adding parasites to the human foreskin fibroblasts (HFFs). No significant reduction in invasion efficiency was observed (unpublished data); hence, binding of antibodies to RON4 either does not inhibit the invasion process or the titer of the antibodies used was insufficient to exert an effect.

IIF of invading wild-type parasites using mAb CL22 and mAb B3.90 to detect TgAMA1 confirmed the reported surface localization of TgAMA1 on invading parasites and showed a marked concentration on the posterior region up to the MJ (Figure 5A and 5B). Co-staining with anti-RON4 showed overlap of the two signals at the MJ (Figure 5C and 5D) although the majority of TgAMA1 was not coincident with RON4. Nevertheless, rotation of the three dimensional rendering makes clear the overlap of TgAMA1 throughout the RON4 ring at the MJ (Figure 5E–H). While these results show some co-localization of RON4 and TgAMA1 at the MJ, the majority of TgAMA1 is clearly posterior to this position.

**Figure 5:**
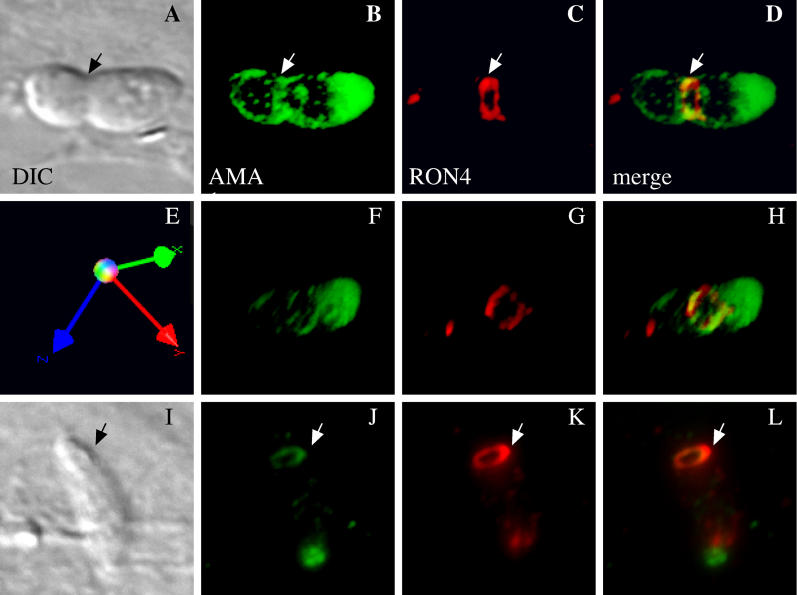
Localization of TgAMA1 and RON4 During Invasion Tachyzoites were allowed to invade following a potassium shift as for Figure 4. DICA, E, and I) and deconvolution IIF were then used to image formaldehyde-fixed parasites as described in Figure 3, except that the monolayers were permeabilized at the outset and a mAb specific for the cytoplasmic tail of TgAMA1 (CL22) was used in place of anti-SAG1. The images shown here are three-dimensional reconstructions from forty 0.1-μm sections. (A–D) show a wild-type parasite that is about 40% inside the host cell (the outside portion shows a bright, posterior cap of CL22 staining). (E) shows the axis of rotation for the reconstruction, and the new view of the IIF images present in (B–D) are shown in the corresponding panels (F–H). Images (I–L) show a *ΔAMA1/AMA1-myc* parasite with low surface AMA1 signal (no posterior cap) that is about 80% invaded into its vacuole. The MJ is indicated by an arrow.

The increase in the stoichiometry of RON4-association with TgAMA1 observed with the *ΔAMA1/AMA1-myc* parasites suggested that their association might be more apparent in IIF using these parasites. This line is fully invasion-competent, yet it has, on average, only ~10% of the wild-type amount of TgAMA1 [23]. Hence, the overall TgAMA1 staining should be less, but that which is observed may be concentrated in regions on the surface most critical for invasion. Repeating the IIF on invading *ΔAMA1/AMA1-myc* parasites confirmed the reduced level of TgAMA1 staining relative to wild-type but revealed that approximately one in twenty of these parasites have a level that is substantially lower than the average (i.e., <<10% of wild-type) (Figure 5I and 5J). This reduction could be due to loss of one or more copies of the *TgAMA1*-*myc* sequence or exhaustion of the store of TgAMA1 during a previous, abortive invasion step. Regardless, examination of these parasites showed a distinct concentration of TgAMA1 at the MJ that precisely co-localized with RON4 (Figure 5I–5L). Together with the immunoprecipitation data above, these results clearly indicate that TgAMA1 and RON4 form a complex (likely with RON2 and TwinScan 4705) that tracks with and, in the case of RON4, defines the MJ in invading parasites.

### Analysis of RON4 Localization Following Ionophore-Induced Egress

Egress involves many of the same phenomena as invasion, including the constriction and an apparent MJ. To address whether the complex identified above might also be present in exiting parasites, we repeated the IIF using ionophore-induced egress [34]. Figure 6B shows that RON4 is indeed detected in rings coincident with constriction as parasites pass through either the PV or the host plasma membrane (solid arrows) as well as at the extreme apical tip of the parasite (open arrow) and in the collapsed parasitophorous vacuole. The ring structure was not detected in the absence of detergent permeabilization (unpublished data), suggesting it is located on the cytoplasmic side of the host cell plasma membrane. TgAMA1 on the apical surface of parasites that protrude from the infected cell is readily detected without detergent permeabilization using mAb B3.90 (unpublished data). Following triton permeabilization, the TgAMA1 staining extends below the constriction observed by differential interference contrast (DIC) light microscopy and can be detected adjacent to and occasionally intersecting with the RON4 ring (Figure 6C and 6D). TgAMA1 is not superimposable throughout the circumferential RON4 staining, contrary to what was observed during invasion.

**Figure 6:**
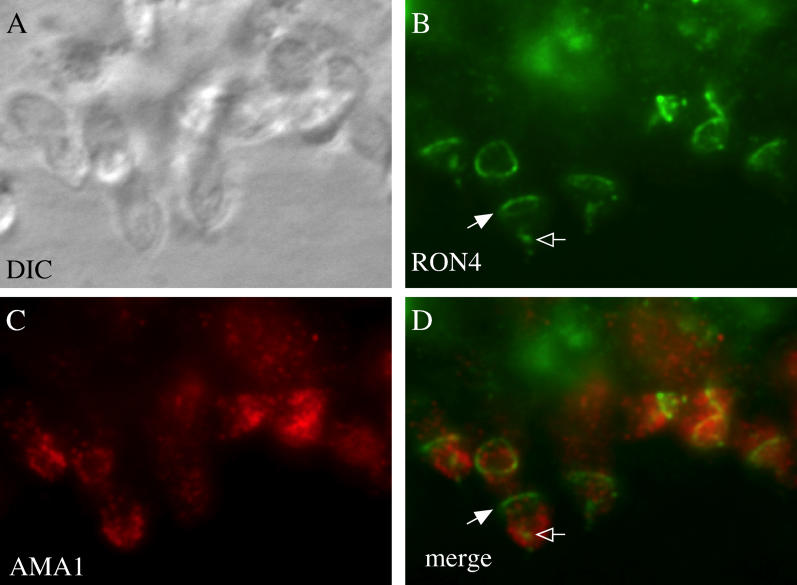
Localization of RON4 and TgAMA1 During Egress DIC (A) and IIF (B, C, and D) were used to image wild-type tachyzoites fixed with formaldehyde one minute after calcium-ionophore treatment to induce egress, and permeabilized with triton X-100. (B) and (C) show staining with anti-RON4 (FITC) and anti-TgAMA1 (Texas Red), respectively. (D) is the merged image. Antibody and IIF details are as for Figure 4.

To address the role of TgAMA1 in egress, *ΔAMA1/AMA1-myc* parasites were grown in the presence of Atc to suppress TgAMA1 levels, and then treated with calcium-ionophore to induce egress*.* Interestingly, unlike the impairment in invasion, there was no effect on egress under these conditions and RON4 localization to rings in the exiting parasites was unaffected (unpublished data). These results indicate that RON4 can function independently of TgAMA1, and that TgAMA1 is not essential for egress. RON4 may be recruited to the MJ wherever the parasites transit membranes, whereas TgAMA1 is apparently required only for invasion.

### Localization of RON4 at the Apical Tip of Parasites in Intimate Contact with Host Cells

Following tetracycline treatment, the majority of *ΔAMA1/AMA1-myc* parasites will attach to host cells but fail to invade [23]. This is associated with a defect in release of certain rhoptry proteins into the host cell based on studies of the well-characterized ROP1 and ROP2/3/4 markers. We asked whether RON4 secretion was also disrupted in the invasion-defective parasites. IIF using antibodies to RON4 showed that for the attached but un-invaded parasites RON4 is secreted to the apical tip of the parasite (Figure 7) even though no TgAMA1 is detectable (unpublished data). This RON4 staining, however, is generally amorphous and bleb-like with no formation of a ring-like MJ. In rare instances, parasites with nascent RON4 rings are observed in the absence of detectable AMA1 although such parasites appear to be severely retarded in their invasion based on the very short distance they have penetrated into the host cells, despite being given an extended time for invasion. Hence, it appears that TgAMA1 is necessary both for the formation and subsequent movement of the MJ complex.

**Figure 7:**
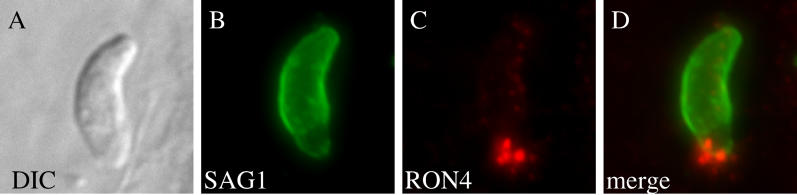
RON4 localization in *ΔAMA1/AMA-myc* Tachyzoites Following Tetracycline Repression and Abortive Invasion DIC (A) and deconvolution IIF (B–D) were used to image formaldehyde-fixed cultures. Images represent a projection through ten 0.2-μm sections. Intracellular *ΔAMA1/AMA-myc* tachyzoites were grown in Atc-containing media for 30 hr, syringe-released and then synchronized for invasion using a potassium shift as described in the methods. After allowing invasion for 20 min, the cover-slips were fixed and processed for IIF without detergent permeabilization. SAG1 staining ([B] FITC) shows an extracellular parasite in contact with the HFF monolayer. (C) shows the RON4 (Texas Red staining). (D) is a merge of (B) and (C).

### RON4 May Be an Essential Protein

To further investigate the function of RON4, attempts were made to delete the *RON4* gene. A knock-out vector was constructed to replace the entire *RON4* gene with the hypoxanthine-xanthine-guanine phosphoribosyl transferase (HXGPRT) selectable marker. *RHΔHXGPRT* tachyzoites were transfected with a linearized knock-out cassette and carried through mycophenolic acid/xanthine selection. Despite several attempts, PCR analysis of genomic DNA isolated from drug-resistant populations indicated that the vector had not replaced the endogenous *RON4* gene. Although a negative result in such experiments is not definitive, we have been successful in knock-out attempts with many other genes and in at least one of the cases where such attempts have failed [18], subsequent studies confirmed the gene to be essential [23]. Although such negative results are not definitive, they are consistent with the *RON4* gene being essential, and suggest that RON4 is an indispensable component of the MJ complex created by these obligate intracellular parasites.

## Discussion

In this report, we describe the coordinated secretion of rhoptry necks and micronemes to form a complex of apparently four proteins, TgAMA1, RON4, RON2 and Ts4705, at least of two of which interact at the MJ of invading parasites. LeBrun et. al. have obtained very similar findings to these, i.e., RON4 is at the moving junction as is part of a stable complex with RON2 and Ts4705[35]. Additional RON proteins have been identified as part of a rhoptry proteome analysis [27] leaving open the possibility that these, and perhaps other proteins, also contribute to the MJ complex but, if so, they do not appear to form a stable association with the TgAMA1-associated complex described here.

TgAMA1 and RON4 co-localize with the MJ at the ring of contact between parasite and host plasma membranes. The MJ is a boundary that excludes antibodies from entering the nascent PV, and the fact that RON4 can be detected at the MJ in the absence of detergent permeabilization indicates that it is on the exterior of the MJ both with respect to the parasite and host plasma membranes and the antibody-excluding portion of the MJ. Some of the MJ proteins are likely below this antibody exclusion zone and/or buried beneath the parasite or host cell membranes [4]. This may ultimately prove to be the location of the RON2 and Ts4705 proteins.

In published electron micrographs of *Plasmodium* [5] and *Toxoplasma* caught in the act of invading red blood cells, which *Toxoplasma* can do in vitro [6], the MJ is associated with electron-dense structures of unknown composition and function (although the cytosolic MCP-1 protein may be involved in the *Plasmodium* parasite [36]). In *Plasmodium*, the electron-dense ring is associated with clearance of the merozoite surface coat as the parasite enters the red blood cell [32]. A similar phenomenon is observed in *Toxoplasma* with GPI-anchored surface antigens, such as SAG1, but only if bivalent antibodies are used to loosely cross-link the proteins [5,32,37]. These observations suggest a model whereby the MJ serves as an area of continuous attachment and detachment of the two parallel membranes that somehow, as the two membranes pass through, sieves out selected membrane proteins [38]. This sieving process serves to push toward the rear or even completely remove parasite ligands like MIC2 that are transiently engaged with host receptors and thus explaining the lack of close association between the parasite and host membranes anterior of the MJ [29,39]. The absence of MIC2 in front of the MJ also argues against continued micronemal secretion after invasion has commenced although we cannot exclude the possibility that selected microneme contents (like TgAMA1) somehow continue to be secreted. It seems more likely that the presence of TgAMA1 in front of the MJ is due to the MJ specifically allowing excess TgAMA1 molecules that are not needed for MJ formation (as well as all GPI-anchored surface proteins) to pass through the sieving complex.

Our data can be integrated with the existing literature to produce the model shown in Figure 8 emphasizing the coordinated secretion and migration of TgAMA1 (green) and RON proteins (red). Micronemes are shown discharging through the rhoptry neck as has been suggested from the restricted access to the apical surface of the parasite [40] and based on the redistribution of PfAMA1 from micronemes to rhoptries in *Plasmodium* schizonts [20]. Secretion of RON4 follows the reorientation of the parasite to put its apical, secretory end in contact with the host membrane; this coincides with the formation of an exclusionary junction between the parasite and host cell plasma membranes. In addition to formation of the junction, one of the earliest events in invasion is the injection of rhoptry bulb proteins into the host cytoplasm where they are transiently detected in “evacuoles” [41]. After this initial stage, the parasite moves into the host cell apparently driven by migration of the MJ to the posterior end. This stage is also associated with the creation of a PV in front of the invading parasite. Once the parasite has fully entered and the PV is essentially complete, a residual focus of RON4 remains exposed on the host cell surface at the junction of PVM and host plasma membrane. This RON4 focus is lost once the PV detaches from the host plasma membrane. This is consistent with the observation of Suss-Toby et al. [3] who demonstrated a delay in the pinching off and separation of the PVM from the host plasma membrane, analogous to the constricted pit stage of receptor-mediated endocytosis. During this penultimate stage, the PVM is topologically just a very deep invagination of the host plasma membrane although the PV interior is inaccessible to macromolecules like antibodies throughout the entire process. Removal or dissociation of the RON4-containing proteinaceous cap may represent the limiting step for membrane fission and final separation of the PV.

**Figure 8:**
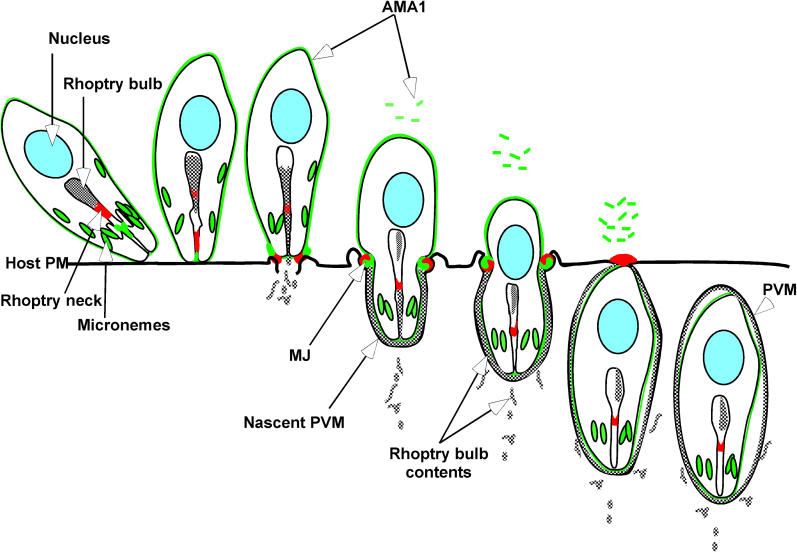
Secretion and Redistribution of TgAMA1 and RON4 During Invasion In this schematic representation, TgAMA1 (green) is secreted to the surface of parasites as they glide across the host surface. Upon reorientation, the amount of TgAMA1 on the surface is increased. A tight association with the host cell membrane is established with the secretion of RON4 (red), and rhoptry bulb constituents (grey) are detected in e-vacuoles within host cytoplasm. The MJ is established and either microneme secretion ceases or some surface proteins are able to pass through the MJ (e.g., TgAMA1, as is known for the GPI-anchored surface antigen, SAG1). RON4 migrates toward the posterior end of the parasite as a discrete ring at the constricted interface with the host cell membrane. TgAMA1 is distributed non-uniformly across the parasite surface on both sides of the MJ with a circumferential concentration at the RON4 ring. The ring containing RON4, TgAMA1, and likely RON2 and Ts4705 migrates the length of the parasite until the parasite is fully enveloped within the parasitophorous vacuole. RON4, but not TgAMA1, is then found on the host cell surface at the junction of the PV and host PM. Release of this MJ complex from the host PM allows the release of the parasite-containing PV into the host cytoplasm.

MIC2 could play a role in forming the MJ complex, but apart from its posterior cap distribution, it shows no specific association with the MJ in IIF experiments (unpublished data; [42]). It is possible that, as with TgAMA1 on wild-type parasites, the failure to specifically detect an accumulation of MIC2 at the MJ by IIF could be due to the strong, distributed signal across the parasite surface behind the MJ. As they stand, however, the above observations suggest that the TgAMA1/RON4 complex at the MJ functions independently of MIC2.

One implication of our model is a slight elaboration of previous models for sequential organelle-secretion during invasion. These were based, in part, on the analysis of rhoptry bulb proteins and held that micronemes release first, then rhoptries and lastly dense granules [1]. Our data argue for the simultaneous secretion of micronemes and at least the rhoptry necks; the rhoptry bulbs may indeed come later. Release of the neck proteins may be activated by the same calcium-responsive pathways controlling microneme secretion [43], and the two may even be physically connected if the micronemes discharge via the rhoptry necks, as previously speculated [14,40].

Our model leaves open how TgAMA1 and the MJ complex link to the cytoskeletal machinery. The fact that invasion is inhibited by Cytochalasin-D treatment clearly indicates a role for actin in the entry process [44,45], and myosin-A has also been implicated [7,46], but whether this reflects a role for these molecules in MJ movement or some other aspect of invasion (e.g., the posterior migration of the MIC2 complex) has yet to be determined. The type-1 transmembrane topology of TgAMA1 and multiple transmembrane domains of RON2 suggest one or other of these could bridge the MJ to cytoskeletal motors. Other type-1 microneme proteins, such as MIC2 in *Toxoplasma* and TRAP in *Plasmodium*, bridge to actin through aldolase [29,47,48] but neither actin nor aldolase were found specifically associated in our TgAMA1- or RON4-immunoprecipitations suggesting that if such a link is involved, it is not stable enough to be detected in these conditions. This suggests an indirect or different linkage to the cytoskeletal motor is involved in MJ vs. MIC2 migration.

The differences in linkage may also be reflected in the parasites speed when gliding vs. invading: gliding motility by *Toxoplasma* tachyzoites is an order of magnitude faster than invasion [49,50]. Likewise, analysis of *Plasmodium* sporozoite motility in vivo shows a major difference in speed during gliding across or even through hepatocytes vs. invasion into a vacuole [51]. The observation that RON4 rings fail to organize and migrate in the absence of TgAMA1 and yet normal gliding motility is unaffected by the absence of this protein [23] further indicates that invasion and gliding are very distinct processes.

RON4 but not TgAMA1 was associated with the MJ during ionophore-induced egress indicating that RON4 can function independently of TgAMA1. Consistent with this, IIF on the bradyzoite stages present in tissue cysts from a chronically infected mouse brain, revealed RON4 in the rhoptry necks and lumenal face of the cyst wall, but no TgAMA1 (unpublished data). This suggests that bradyzoites may utilize an alternative protein to coordinate with RON4 and undergo invasion. MIC7 and MIC9 are bradyzoite-specific microneme proteins that could fulfill such a role [52,53].

BLAST analysis reveals homologues in the predicted protein database of *Plasmodium falciparum* (http://plasmodb.org) for each of the TgAMA1-associated proteins: RON2\′s homologue is Pf14_0495 (e-value ~10^−44^), RON4 corresponds to Pf11_0168 (e-value ~10^−9^), and Ts4705 corresponds to MAL8P1.73 (e-value ~10^−9^). The *Plasmodium* homologs for RON2 and RON4 are predicted to be somewhat larger in size, but overall these proteins have similar predicted structural topology except that the homologue for Ts4705 lacks a predicted signal sequence; it does, however, have a strong hydrophobic stretch near the N-terminus of the predicted protein that may serve this function in reality. Homologues for RON2 and RON4 are also found in expressed sequence tag sequences for *Neospora caninum*. Consistent with this, our RON4 antisera detect distinct rings at the moving junction of invading *N. caninum* (unpublished data). The microneme protein TgAMA1 is highly conserved among Apicomplexa and the finding that all of the associating rhoptry neck proteins are also conserved strongly suggests that coordinated secretion from these two compartments is a common mechanism whereby the components of the moving junction are assembled at the interface between host and parasite plasma membranes.

In addition to its clear similarity to Pf14_0495, RON2 also has weak BLAST similarity (e-value ~10^−8^) to another published protein in *P. falciparum*, PfRhopH1 [54]. This latter protein is part of a complex of three high molecular weight proteins within the rhoptry necks of *P. falciparum* [55]. None have yet been found at the MJ but the homology to RON2 and their presence in the necks makes this possibility tantalizing. Although the other two partners of PfRhopH1, PfRhopH2 and PfRhopH3, have no detectable homologues in the *Toxoplasma* genome, it could be that they represent further components of the MJ and that sequence drift prevents identification through simple BLAST analysis. The different host and cellular niches of these parasites could easily produce the kind of evolutionary pressure necessary for such rapid drift. Of particular interest is the extraordinarily broad host range of *Toxoplasma* compared to most of the other Apicomplexa. It is tempting to speculate that specific adaptations of the MJ might be involved, e.g., in providing the parasite its own anchor in the host cell membrane and thus essentially all of the machinery necessary to invade without demanding anything of the host cell except its membrane. Which aspects of *Toxoplasma*'s MJ constituents are unique will be interesting to explore as an explanation for this parasite's universal taste.

## Materials and Methods

### Parasite culture.

*T. gondii* tachyzoites lacking the *RHΔHXGPRT* gene were used for generation of lysates and the immunofluorescence analysis shown here [56,57]. The tetracycline-repressible TgAMA1-expressing parasite line, *ΔAMA1/AMA1-myc*, and parental line retaining the endogenous *TgAMA1* gene, *AMA1/AMA1-myc*, were derived in the TgTA line [11,23]. Immunofluorescence localization analysis was also carried out in the Prugniaud strain [57] and did not differ from the results described here for the *RHΔHXGPRT* strain. Tachyzoites were grown in monolayers of HFF, exactly as previously described [18]. The *ΔAMA1/AMA1-myc* and parental *AMA1/AMA1-myc* were grown in media containing 50 μg/ml mycophenolic acid, 50 μg/ml xanthine and 20 μM chloramphenicol [46,58]. *ΔAMA1/AMA1-myc* parasites were depleted of TgAMA1-myc by adding fresh media containing 1.0 μg/ml (Clontech, Palo Alto, California, United States) to HFF monolayers infected at an MOI of 5, 1 hr prior to changing the media. Parasites were then grown for 24–30 hr in the presence of Atc and harvested by syringe-release. Under these conditions TgAMA1-myc was reduced to ~0.25% of constitutive TgAMA1 amounts as assessed by immunoblot analysis.

### Immunological procedures.

The mAbs specific for TgAMA1 were CL22 [18] and B3.90 [19]. For immunoprecipitation of TgAMA1, these monoclonal antibodies were bound and coupled using dimethyl pimelimidate to fastflow Protein G sepharose (Amersham Biosciences, Little Chalfont, United Kingdom) [59].

Tachyzoite lysates were generated by extracting 5 × 10^9^ extracellular parasites in 10 ml of TEN buffer (50 mM Tris [pH 8.0], 5 mM EDTA, 150 mM NaCl) containing RIPA detergents [1% NP40, 0.5% Deoxycholate, 0.01% SDS, and Complete protease inhibitor, (Roche Diagnostics, Mannheim, Germany)] on ice for 30 min and removing insoluble material by centrifugation at 3,000 × g for 20 min.

These extracts were incubated with the indicated antibodies coupled to protein G sepharose for 4 hr at 4 °C, followed by three washes (15-min each) in RIPA buffer and three washes in Tris-buffered saline (TBS; 50 mM Tris [pH 8.0], 150 mM NaCl). Bound polypeptides were eluted with 0.1 M triethylamine [pH 11.5] (using five successive elutions), and lyophilized to concentrate the eluate and remove the triethylamine. The 10^6^ parasite equivalents were fractionated by SDS-PAGE for immunoblot analysis, or, similarly, 10^8^ parasites for Coomassie brilliant blue staining.

### Cross-linking of surface proteins.

A potassium buffer shift was used to synchronize invasion as described elsewhere [31]. Briefly, tachyzoites were harvested, washed, and resuspended in Endo Buffer (10 mM K_2_SO_4_, 2.5 mM Mg_2_SO_4_, 1 mM glucose, 5 mM Tris [pH 8.2], and 3.5 mg/ml BSA). They were then added in a volume of 10 ml to HFF monolayers in 150-mm dishes and allowed to settle at 37 °C for 15 min. The Endo buffer was removed, and 10 ml of PBS, at 37 °C, containing 50 μM DTSSP, was added to simultaneously initiate invasion and cross-linking. This incubation was carried out for 20 min at 37 °C. The HFF monolayers were washed twice with TBS to remove and quench the cross-linker. These monolayers with attached and invaded parasites were harvested, denatured by heating to 95 °C for 5 min in 1% SDS, then equilibrated in RIPA buffer, and processed as described for immunoprecipitation analysis. Under these conditions, the DTSSP cross-linker reduced invasion by ~25% as assessed by SAG1 antibody accessibility.

### Protein analysis and mass spectrometry.

Individual bands from Coomassie stained SDS-PAGE gels were excised, treated with trypsin, and extracted for LC-MS/MS analysis. This analysis was performed at the Stanford University mass spectrometry facility (http://mass-spec.stanford.edu/), and the peptide fragment fingerprint data were subjected to a combined database search of the TwinScan-predicted proteins from the *Toxoplasma* genome database (ToxoDB; http://ToxoDB.org), and the NCBI-nr protein database by using the Bioworks MS/MS ion search program(www.thermo.com/) and Xtandem search included in the Scaffold analysis software (www.proteomesoftware.com). Peptides were ranked by an X-corr >3.2 and a D-CN >0.25, and the individual spectra examined for ion coverage and overall quality.

### Nucleic acid techniques.

The generation of complete coding region cDNAs for RON2 and RON4 were as described previously, as was the use of recombinant versions of these proteins to produce antibodies in mice [27].

Constructs for the disruption of RON4 were generated using *HXGPRT* selection and the vector *pMini-GFP.ht* [60]. For the knockout construct, a PCR fragment from genomic bases −3941 to −1939, relative to the start codon, were digested with Kpn and Apa1, and ligated into *pMini-GFP.ht* similarly cut. Into the resulting construct, a PCR fragment of genomic bases 12,053–14,276 (again relative to the ATG start codon) digested with Not1 and SpeI was cloned. The primers used to expand the flanking regions were 5′-CGGGGTACCCCAATCAAAATCCGCAATAGCC-3′, and 5′-CGGGTGCACCAGGTGACCCGTCCATAC −3′ for the upstream flank and 5′-GGACTAGTTTGCCTTGTTTCGCCTTAC-3′, and 5′-AAGCGGCCGCTGTTTCCCTTTGAACTCTGCCAC-3′ for the downstream flank.

The resulting vector *pKORON4* was linearized with NotI, and 50 μg of DNA was electroporated into *RHΔHXGPRT* strain parasites by standard methods, and selection of *HXGPRT* parasites was performed as previously described [58]. Four independent transformations were carried out. Ten days after transformation and MPA/xanthine selection, DNA was isolated from selected populations and PCR used to screen for the presence of knockout parasites. An *HXGPRT*-internal primer (5′-GTGGCGATTCTCATCGACTT-3′) and a primer representing the region upstream of *RON4* (5′- CTTCTTCGGTTCCTCGTTAG-3′) were used for PCR detection of integration into the upstream flanking region.

### Microscopy.

Analysis of tachyzoite-invasion was performed following potassium buffer shift to synchronize invasion essentially as described above. Specifically, 10^6^ parasites in Endo buffer were added onto HFF monolayers grown on 20-mm cover-slips in 24-well plates and incubated for 15 min at 37 °C to settle and contact the monolayer. Invasion was initiated by exchanging the buffer to HBSS, supplemented with 1% FBS at 37 °C followed by incubation for either 1 min to capture partially invaded parasites, or 20 min for a predominantly fully invaded population. Tachyzoites were washed in PBS, and fixed with 3.5% formaldehyde in 150 mM phosphate buffer [pH 7.2], washed and processed for indirect immunofluorescence as described previously [18]. Alternatively, fixation was carried out using 100% methanol at −20 °C for 2 min.

Specific antibody staining was developed with appropriate Alexa488- or Alexa594-secondary antibodies (Molecular Probes, Eugene Oregon, United States). Phase and fluorescence images were captured at 100× on an Olympus BX60 and a Hamamatsu Orca100 CCD, and were pseudo-colored and merged using Image pro-plus 2.0 software (Mediacybernetics, Silver Spring, Maryland, United States). Where indicated, serial Z-stacks images were collected at 100× on a motorized Zeiss (Thornwood, New York, United States) Axiovert 200M equipped for DIC light microscopy. In these cases, fluorescence images were captured with a Hamamatsu Orca2 CCD camera (Hamamatsu, Hamamatsu City, Japan) and were deconvolved by using an iterative algorithm and calculated point spread function, pseudo-colored, and merged using Openlab 4.02 and Velocity 3.01 software (Improvision, Lexington, Massachusetts, United States).

## Abbreviations

AAP: TgAMA1-associating protein
AMA1: apical membrane antigen 1
Atc: anhydrotetracycline
DIC: differential interference contrast
DTSSP: 3,3′-Dithiobis (sulfosuccinimidylpropionate)
FITC: fluorescein isothyocyante
HFF: human foreskin fibroblasts
IIF: indirect immunofluorescence
LC-MS/MS: liquid chromatography-electrospray ionization-ion trap mass spectrometry
mAb: monoclonal antibody
MJ: moving junction
PVM: parasitophorous vacuolar membrane
PV: parasitophorous vacuole
HXGPRT: hypoxanthine-xanthine-guanine phosphoribosyl transferase
RON: rhoptry neck protein
SAG1: surface antigen 1
TgACT1: Toxoplasma actin
TgAMA1: T. gondii version of AMA1
